# Associations of sNfL with clinico‐radiological measures in a large MS population

**DOI:** 10.1002/acn3.51704

**Published:** 2022-11-25

**Authors:** Elias S. Sotirchos, Kathryn C. Fitzgerald, Carol M. Singh, Matthew D. Smith, Maria Reyes‐Mantilla, Carrie M. Hersh, Megan H. Hyland, Ryan Canissario, Sarah B. Simmons, Georgina Arrambide, Xavier Montalban, Manuel Comabella, Robert T. Naismith, Min Qiao, Lauren B. Krupp, Jacqueline A. Nicholas, Katja Akgün, Tjalf Ziemssen, Richard Rudick, Elizabeth Fisher, Robert A. Bermel, Ellen M. Mowry, Peter A. Calabresi

**Affiliations:** ^1^ Present address: Department of Neurology Johns Hopkins University School of Medicine Baltimore Maryland USA; ^2^ Biogen Cambridge Massachusetts USA; ^3^ Lou Ruvo Center for Brain Health Cleveland Clinic Las Vegas Nevada USA; ^4^ Department of Neurology University of Rochester Medical Center Rochester New York USA; ^5^ Mellen Center, Neurological Institute, Cleveland Clinic Cleveland Ohio USA; ^6^ Department of Neurology and Centre d'Esclerosi Múltiple de Catalunya Vall d'Hebron Hospital Universitari, Universitat Autònoma de Barcelona Barcelona Spain; ^7^ Department of Neurology Washington University in St. Louis St. Louis Missouri USA; ^8^ Department of Neurology New York University New York City New York USA; ^9^ OhioHealth Multiple Sclerosis Center Riverside Methodist Hospital Columbus Ohio USA; ^10^ Center of Clinical Neuroscience, Department of Neurology University Clinic Carl‐Gustav Carus Dresden Germany; ^11^ (formerly) Biogen Cambridge Massachusetts USA

## Abstract

**Objective:**

Evaluation of serum neurofilament light chain (sNfL), measured using high‐throughput assays on widely accessible platforms in large, real‐world MS populations, is a critical step for sNfL to be utilized in clinical practice.

**Methods:**

Multiple Sclerosis Partners Advancing Technology and Health Solutions (MS PATHS) is a network of healthcare institutions in the United States and Europe collecting standardized clinical/imaging data and biospecimens during routine clinic visits. sNfL was measured in 6974 MS and 201 healthy control (HC) participants, using a high‐throughput, scalable immunoassay.

**Results:**

Elevated sNfL levels for age (sNfL‐E) were found in 1238 MS participants (17.8%). Factors associated with sNfL‐E included male sex, younger age, progressive disease subtype, diabetes mellitus, impaired renal function, and active smoking. Higher body mass index (BMI) was associated with lower odds of elevated sNfL. Active treatment with disease‐modifying therapy was associated with lower odds of sNfL‐E. MS participants with sNfL‐E exhibited worse neurological function (patient‐reported disability, walking speed, manual dexterity, and cognitive processing speed), lower brain parenchymal fraction, and higher T2 lesion volume. Longitudinal analyses revealed accelerated short‐term rates of whole brain atrophy in sNfL‐E participants and higher odds of new T2 lesion development, although both MS participants with or without sNfL‐E exhibited faster rates of whole brain atrophy compared to HC. Findings were consistent in analyses examining age‐normative sNfL Z‐scores as a continuous variable.

**Interpretation:**

Elevated sNfL is associated with clinical disability, inflammatory disease activity, and whole brain atrophy in MS, but interpretation needs to account for comorbidities including impaired renal function, diabetes, and smoking.

## Introduction

Multiple sclerosis (MS) is an immune‐mediated, inflammatory disorder of the central nervous system. Despite being classically considered a demyelinating disorder, neuro‐axonal injury occurs early in the disease course and represents the pathologic substrate for permanent neurological disability in people with MS (PwMS).^1^ In clinical practice, disease monitoring in PwMS is performed by clinical evaluation and use of conventional magnetic resonance imaging (MRI) measures, including new T2 lesions and/or presence of T1 post‐gadolinium (Gd)‐enhancing lesions. Notably, these conventional MRI measures assess for the presence of inflammatory disease activity rather than neuro‐axonal injury, and biomarkers identifying the latter are an important unmet need in MS and other neurological conditions.

Neurofilaments are neuron‐specific cytoskeletal proteins that are released into the extracellular space following neuro‐axonal damage, and have thus been proposed as putative biomarkers of neuro‐axonal injury in multiple neurological diseases, including MS.^2^ Neurofilament light chain (NfL) has particularly been shown to be a promising biomarker because of its high solubility. The application of newer immunoassays has enabled the measurement of the low concentrations of NfL in blood (usually serum) with high accuracy and reproducibility, and increased NfL levels have been found in blood in several neurological disorders with underlying neuro‐axonal degeneration, including MS.^3^, ^4^

In MS, there is evidence that serum NfL (sNfL) levels correlate with CSF NfL, are associated with clinico‐radiological inflammatory disease activity, disability progression, and brain atrophy, and are modulated by disease‐modifying therapies (DMTs).^3^, ^5^, ^6^, ^7^, ^8^, ^9^ However, the vast majority of studies in which sNfL has been studied in MS have been single‐center studies or post hoc analyses of clinical trial cohorts, which may lack generalizability. Furthermore, while it is clear that age is a very important factor that needs to be accounted for when interpreting sNfL levels, the effect of comorbidity or varying MS characteristics on sNfL in MS has not been rigorously characterized at scale. Importantly, data support that factors including body mass index (BMI), diabetes mellitus, and impaired renal function may influence sNfL levels.^9^, ^10^, ^11^, ^12^, ^13^ Additionally, most existing studies have been performed using single molecule array (Simoa), which is run on a research platform. In order for sNfL testing to be more readily implemented in the clinical arena, evaluation of high‐throughput assays on routine clinical laboratory platforms is necessary.^14^

In this study, we sought to evaluate associations of sNfL, measured using a high‐throughput scalable immunoassay, with demographics, comorbid conditions, MS clinical characteristics and clinico‐radiological outcomes, in a large, international, real‐world, multi‐center population of PwMS.

## Methods

### Study participants

Data for this study were collected as part of the Multiple Sclerosis Partners Advancing Technology and Health Solutions (MS PATHS) network between November 2016 and May 2021. The design of MS PATHS has been previously described in detail.^15^ Briefly, MS PATHS is an ongoing initiative conducted in 10 MS centers (seven in the United States, three in Europe), that have standardized elements of their clinical practice and collaborated with Biogen to implement a centralized health information exchange architecture for research purposes. MS PATHS was designed around the concept of a learning health system, merging research with ongoing patient care by collecting standardized data during routine clinic visits. Enrollment in MS PATHS is open to individuals with a physician‐confirmed diagnosis of MS (including clinically isolated syndrome [CIS]) seeking care at participating institutions. Additionally, healthy controls (HC) have been recruited at participating healthcare institutions to be demographically representative of the MS cohort with respect to age, sex, and race. Pertinent exclusion criteria for HC included: (1) pregnancy, (2) diagnosis of migraine requiring medication or any other subject‐reported diagnosis of neurological disease or condition, and (3) any subject‐reported diagnosis of an autoimmune disorder. All participants provided written informed consent to collect and share pseudo‐anonymized data; the study was approved by the institutional review board of each participating institution.

### MS performance test

A full list of data elements collected as part of MS PATHS has previously been reported.^15^ Briefly, MS PATHS incorporates a self‐administered iPad‐based patient assessment tool, the MS Performance Test (MSPT), to collect a structured history, patient‐reported outcomes, and an assessment of neuroperformance, using an adaptation of the MS Functional Composite (MSFC) to assess walking speed, manual dexterity, and cognition.^16^ Specific clinical disability outcomes of interest considered in this study include: (1) Patient Determined Disease Steps (PDDS), (2) a 25‐foot walking speed test, similar to the Timed 25‐Foot Walk, (3) a manual dexterity test, similar to the 9‐Hole Peg Test, and (4) a processing speed test, similar to the Symbol Digit Modalities Test. The PDDS is a patient‐reported outcome, adapted from the clinician‐assessed Disease Steps, which has previously been shown to exhibit excellent reproducibility, to be sensitive to disability progression, and to correlate well both cross‐sectionally and longitudinally with the Expanded Disability Status Scale (EDSS).^17^ The electronic adaptation of the MSFC has similarly been validated against the analogous technician‐administered tests and has been shown to exhibit excellent correlations in PwMS.^16^, ^18^, ^19^ Additional data elements, including medical history, medication lists, and physical/laboratory assessments are automatically extracted from the electronic medical record (EMR).

### Biospecimen collection and sNfL measurement

All participants in MS PATHS are also offered the option at each clinic visit to provide blood samples for a bio‐banking sub‐study. Blood samples for the MS PATHS biobanking sub‐study are collected at each study site, according to a standardized MS PATHS protocol.^15^ Briefly, for serum collection, blood is collected by venipuncture in serum separator tubes (SST), mixed by inversion, and allowed to clot for ~30 min at room temperature in an upright position. Within 1 h of collection, SST are centrifuged for 15 min at ~1500 g. Following centrifugation, the serum is aliquoted and frozen immediately at −70°C (±10°C). If a −70°C freezer is not available, samples may be frozen and stored at −20°C until shipped. Samples are shipped on dry ice weekly to central laboratories for long‐term storage at −70°C.

**Figure 1:**
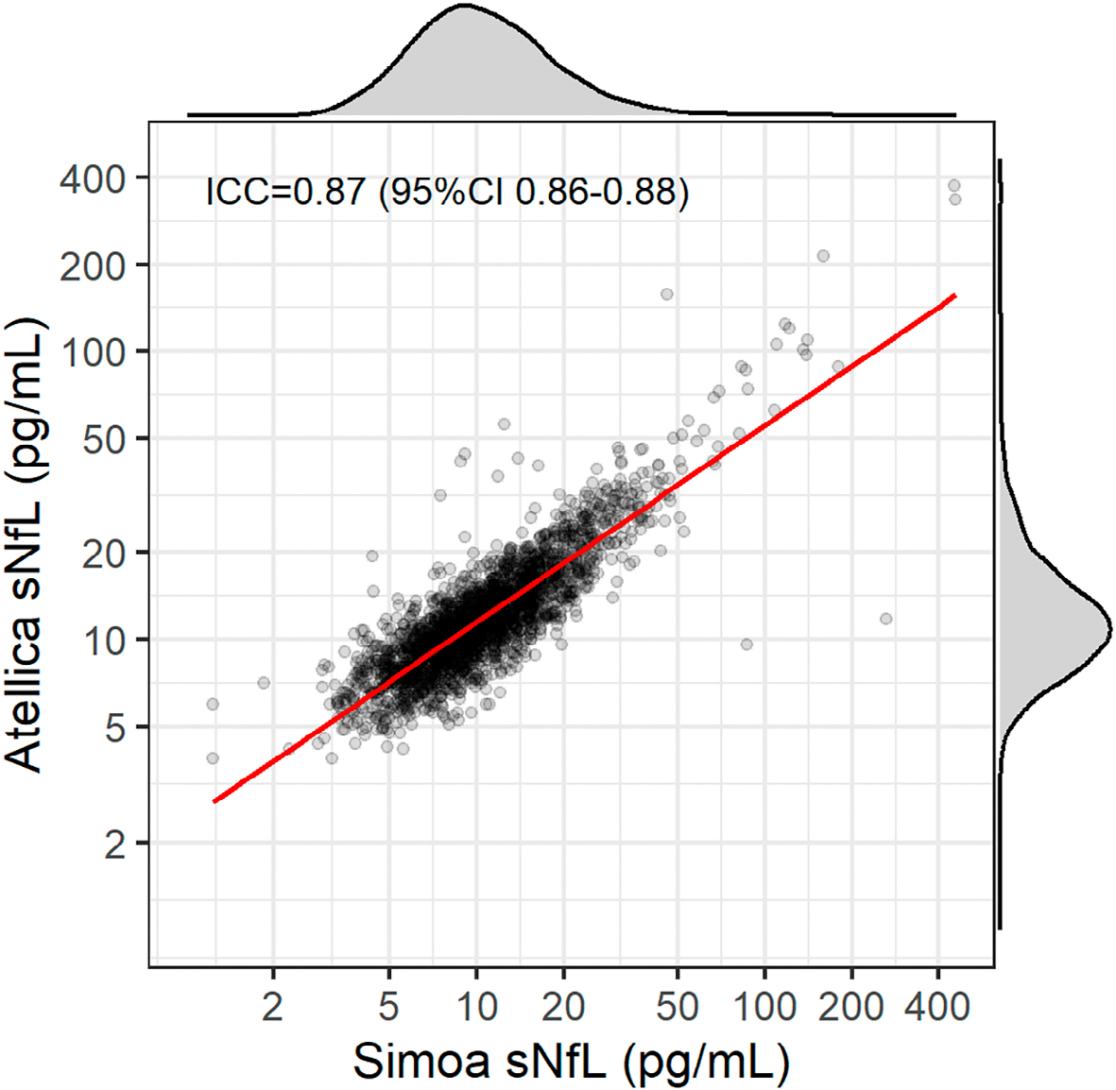
sNfL measured on the Siemens Atellica Platform versus the Quanterix Simoa Platform. Plot of sNfL levels (pg/mL) measured on the Siemens Atellica (y‐axis) and Quanterix Simoa platforms (x‐axis) in *n* = 2143 participants. The overlaid red line corresponds to the ordinary least squares regression line fit. The corresponding marginal densities for each variable are shown in the plot margins. ICC, intra‐class correlation coefficient; CI, confidence intervals.

Serum samples for all MS PATHS biobanking sub‐study participants from the first available timepoint were retrieved for sNfL measurements. Samples were shipped to Siemens Healthcare Laboratory, LLC (Berkeley, CA, USA) on dry ice and upon receipt, samples were stored at −80°C. Prior to analysis, samples were thawed at room temperature and measurements were performed on the Atellica® Solution platform, using a high‐throughput scalable acridinium‐ester immunoassay, which has a range of 3.9 to 500 pg/mL and excellent repeatability.^14^, ^20^, ^21^ All runs included low (~7 pg/mL), medium (~40 pg/mL), and high (340 pg/mL) quality control samples to ensure assay stability over time, with acceptable precision (coefficient of variation 10.2%, 5.4%, and 7.3%, respectively) and no significant change over time. Furthermore, sNfL measurement in different serum aliquots from the same blood draws was performed previously on a Simoa HD‐1 Analyzer for a subset of participants (*n* = 2143), using the Simoa NfL Advantage Assay Kit.^22^ For the Simoa sNfL measurement, the samples were similarly shipped on dry ice to a central laboratory (Quanterix; Billerica, MA, USA) and stored at −80°C prior to thawing and subsequent analysis. sNfL values obtained by the two methods exhibited good correlation and agreement (*r* = 0.85, ICC 0.87; Fig. 1).

### Magnetic resonance imaging

Standardized MRI protocols are utilized in MS PATHS to acquire brain imaging on Siemens 3 T Scanners, as previously described.^15^ Brain MRIs are acquired as clinically indicated, generally occurring annually for monitoring purposes. Radiology report data are extracted from the EMR, including the number of T1 post‐Gd‐enhancing lesions (if a post‐contrast scan was acquired) and the number of new T2 lesions since the prior MRI (if a comparable scan was available), as read by the local radiologists at each institution. Quantitative MRI metrics are also derived from brain MRIs as part of MS PATHS, including brain parenchymal fraction (BPF) and T2 lesion volume. These values are automatically calculated utilizing a software prototype developed jointly by Biogen and Siemens, MSPie (MS PATHS Image Evaluation).^23^ BPF is a normalized measure of whole brain volume, calculated using a combination approach based on segmentation of the brain parenchyma and total intracranial volume in the 3D FLAIR and 3D T1 images. Whole brain atrophy is calculated as the difference in BPF values from two time points. In analyses of a scan–rescan substudy of MS‐PATHS including three MS‐PATHS sites at which 30 MS patients underwent four MRIs on two different Siemens 3 T MRI scanners within 1 week, MSPie BPF estimated by MSPie exhibited excellent reproducibility, with a mean coefficient of variation of 0.18%. T2 lesions are segmented automatically in MSPie based on 3D FLAIR and 3D T1 images using the LeMan‐PV algorithm.^24^

### Statistical methods

Statistical analyses were performed with R Version 4.1.2 (https://www.r‐project.org/). We used sNfL measurements from the MS PATHS HC cohort to derive age‐normative Z‐scores using Generalized Additive Models for Location, Scale, and Shape (GAMLSS).^5^, ^25^, ^26^ We selected the Box‐Cox Cole and Green distribution family as this minimized the Generalized Akaike information criterion. Analyses were performed using the age‐normative sNfL Z‐score as a continuous variable (truncated at the 99.9th percentile), as well as using a pre‐defined Z‐score cut‐off of 1.96 (97.5th centile curve) to define “elevated” (sNfL‐E) versus “normal” sNfL (sNfL‐N), as this is the typical approach used to define reference ranges for clinical laboratory tests.^27^ sNfL values below the lower limit of quantification (LLoQ; 3.9 pg/mL) were replaced by the LLoQ (*n* = 18 MS samples < LLoQ; none of the HC samples).

Furthermore, we also derived age‐normative sNfL Z‐scores using a similar approach, using sNfL measured in the National Health and Nutrition Examination Survey (NHANES), as reported in detail previously.^13^ Given that the previously reported NHANES derived model include age, creatinine, and glycosylated hemoglobin (HbA1c), with creatinine available for a subset of our cohort and HbA1c not available for the vast majority of our cohort, derived age‐normative curves assume a creatinine of 0.79 mg/dL (median value of MS PATHS participants with available creatinine measurements [*n* = 4667] without evidence of chronic kidney disease [i.e., estimated glomerular filtration rate > 60 mL/min/1.73 m^2^]) and HbA1c of 5.22% (median value of non‐diabetics in NHANES).^13^

Age and disease duration were categorized each into five groups based on quintile splits. Estimated glomerular filtration rate (eGFR) was calculated using the creatinine value (if available) closest to the date of blood sampling for sNfL measurement, using the CKD‐EPI 2021 equation incorporating age and sex, omitting race, and was categorized as >90 (“normal”), 60–90 (“mildly impaired), and <60 mL/min/1.73 m^2^ (“moderately to severely impaired”).^28^ Body mass index (BMI) was categorized as <18.5 (“underweight”), 18.5–24.0 (“reference”), 25.0–29.9 (“overweight”), 30.0–39.9 (“obese”), and ≥ 40 (“morbidly obese”). DMT use was categorized as “IFN‐b/GA” (interferon‐beta and glatiramer acetate), oral (teriflunomide, sphingosine‐1‐phosphate inhibitors, and fumaric acid esters), “infusion or immune reconstitution therapy” (anti‐CD20 agents, natalizumab, alemtuzumab, and cladribine), “other or not listed,” and “untreated.” Missing indicator levels were used in multivariable analyses for variables available only for a subset of the cohort (listed in Table 1). While we considered alternative approaches for missing data including imputation or complete case analysis, some key variables were unlikely to be reliably imputed using available predictors (e.g., eGFR without a creatinine measurement), and complete case analysis generally reduces the effective sample size. Furthermore, given that race is largely a social construct and MS PATHS includes sites across the United States and Europe, race in this study may be a surrogate of differing societal dynamics. Therefore, we report results without the inclusion of race as an independent variable. Secondary analyses considered race with similar results observed. Using the PDDS, self‐reported disability was categorized as “mild” (no gait impairment), “moderate” (early gait impairment), and “severe” (assistive device for ambulation or non‐ambulatory).^17^ Neuroperformance domains were transformed to Z‐scores using regression‐based equations derived from a study of adult healthy volunteers (*n* = 517).^29^ T2 lesion volume (log‐transformed) and BPF were converted to Z‐scores (based on the distribution in the MS participants) in order to facilitate interpretation of the results.

**Table 1:** Baseline demographics and clinical characteristics of the MS participants.

	Overall (*N* = 6974)	sNfL‐N, (*N* = 5736)	sNfL‐E (*N* = 1238)
Age (years), mean (SD)	46.9 (12.0)	47.2 (11.5)	45.5 (14.2)
Female, *n* (%)	5017 (72%)	4149 (72%)	868 (70%)
Race, *n* (%)			
White	5751 (82%)	4804 (84%)	947 (76%)
Black	501 (7.2%)	360 (6.3%)	141 (11%)
Other	332 (4.8%)	265 (4.6%)	67 (5.4%)
sNfL (pg/mL), median (IQR)	11.1 (8.4, 14.8)	10.1 (8.0, 12.8)	21.6 (16.6, 28.9)
Brain parenchymal fraction, mean (SD)	0.851 (0.025)	0.851 (0.025)	0.848 (0.028)
T2 lesion Volume (mL), median (IQR)	6.9 (3.2, 14.9)	6.3 (3.0, 13.7)	10.4 (4.9, 22.3)
Disease duration (years), median (IQR)	12.2 (5.9, 20.5)	12.4 (6.1, 20.4)	11.4 (4.7, 21.1)
MS subtype, *n* (%)			
CIS/RRMS	4558 (65%)	3866 (67%)	692 (56%)
Progressive MS	2059 (30%)	1597 (28%)	462 (37%)
Self‐reported disability status, *n* (%)			
Mild	3860 (59%)	3308 (61%)	552 (48%)
Moderate	1295 (20%)	1052 (19%)	243 (21%)
Severe	1390 (21%)	1037 (19%)	353 (31%)
DMT class			
None	940 (13%)	710 (12%)	230 (19%)
IFN‐b/GA^1^	1373 (20%)	1153 (20%)	220 (18%)
Oral^1^	2213 (32%)	1921 (33%)	292 (24%)
Infusion/IRT^1^	1729 (25%)	1373 (24%)	356 (29%)
Other^2^ or Unknown	719 (10%)	579 (10%)	140 (11%)
eGFR (mL/min/1.73 m^2^), *n* (%)			
>90	3342 (48%)	2743 (48%)	599 (48%)
60–89	1226 (18%)	1003 (17%)	223 (18%)
<60	99 (1.4%)	50 (0.9%)	49 (4.0%)
Body mass index, *n* (%)			
<18.5	105 (1.5%)	72 (1.3%)	33 (2.7%)
18.5–24.9	2213 (32%)	1768 (31%)	445 (36%)
25–29.9	1876 (27%)	1574 (27%)	302 (24%)
30–39.9	1830 (26%)	1521 (27%)	309 (25%)
>40	427 (6.1%)	352 (6.1%)	75 (6.1%)
Diabetes mellitus, *n* (%)	434 (6.2%)	321 (5.6%)	113 (9.1%)
Smoking status, *n* (%)			
Non‐smoker	5676 (81%)	4685 (82%)	991 (80%)
Current smoker	1207 (17%)	975 (17%)	232 (19%)

Characteristics available for a subset of the cohort included: race (*n* = 6584), disease subtype (*n* = 6627), disease duration (*n* = 6355), PDDS (*n* = 6545), DMT class (*n* = 6255), eGFR (*n* = 4667), body mass index (*n* = 6451), diabetes status (5606), smoking status (*n* = 6883), and brain parenchymal fraction/T2 lesion volume (within 365 days of blood sampling; *n* = 3514).

sNfL‐E, elevated sNfL; sNfL‐N, “normal” sNfL; SD, standard deviation; IQR, interquartile range; CIS, clinically isolated syndrome; RRMS, relapsing–remitting MS; PDDS, Patient Determined Disease Steps; DMT, disease‐modifying therapy; IFN‐beta, interferon‐beta; GA, glatiramer acetate; IRT, immune reconstitution therapy; eGFR, estimated glomerular filtration rate.

^1^ IFN‐b/GA (IFN‐b *n* = 611; GA *n* = 762), Oral (Fumarate *n* = 1054; S1P inhibitor *n* = 874; Teriflunomide *n* = 285), Infusion/IRT (anti‐CD20 *n* = 943; Natalizumab *n* = 684; Alemtuzumab *n* = 89; Daclizumab *n* = 10; Cladribine *n* = 3).

^2^ A small proportion of participants (*n* = 47) reported use of oral immunosuppressive medications (including mycophenolate, methotrexate, cyclosporine, or cyclophosphamide) or intravenous immune globulin (*n* = 7).

Assessments of the associations of sNfL‐E with the various patient demographics, comorbidity, and clinical characteristics were performed using logistic regression; similar models considered age‐normative sNfL Z‐scores using linear regression. Neuroperformance measures and radiological outcomes were compared between sNfL groups and their associations with age‐normative sNfL Z‐scores were assessed in generalized linear models adjusted for age, sex, BMI, disease duration, disease subtype, DMT class, eGFR category, diabetes mellitus, and smoking status (covariates selected based on the findings in the baseline, cross‐sectional analyses). Self‐reported disability status was compared between sNfL groups using multinomial regression and longitudinal analyses of BPF (dependent variable) were performed using mixed‐effects models including time, sNfL group, and the cross‐product of time and group, as well as random intercepts and random slopes in time to account for within patient correlation, and were similarly adjusted, as previously described.^30^

Analyses were also similarly performed including the age‐normative sNfL Z‐score as a continuous variable, using restricted cubic splines in order to assess for non‐linear relationships; restricted cubic spline models were fit with four knots, placed at the 5th, 35th, 65^th^, and 95th percentile of the age‐normative sNfL Z‐score distribution.^31^ Linear versus spline models were formally compared using likelihood ratio tests; spline models are reported if deviation from linearity was detected (*P* < 0.05).^31^, ^32^

## Results

### Study population

Serum samples were available for 7246 PwMS and 201 HC. Samples from 272 MS participants were excluded due to insufficient quantity of serum in the aliquots (*n* = 204) or excessive hemolysis (*n* = 68), leaving 6974 MS participants with samples available for sNfL measurement. Characteristics of the MS participants and HC are shown in Table 1 and Table S1, respectively. Participant recruitment by site is shown in Table S2.

### Associations of sNfL with demographics, clinical characteristics, and comorbidities

**Figure 2:**
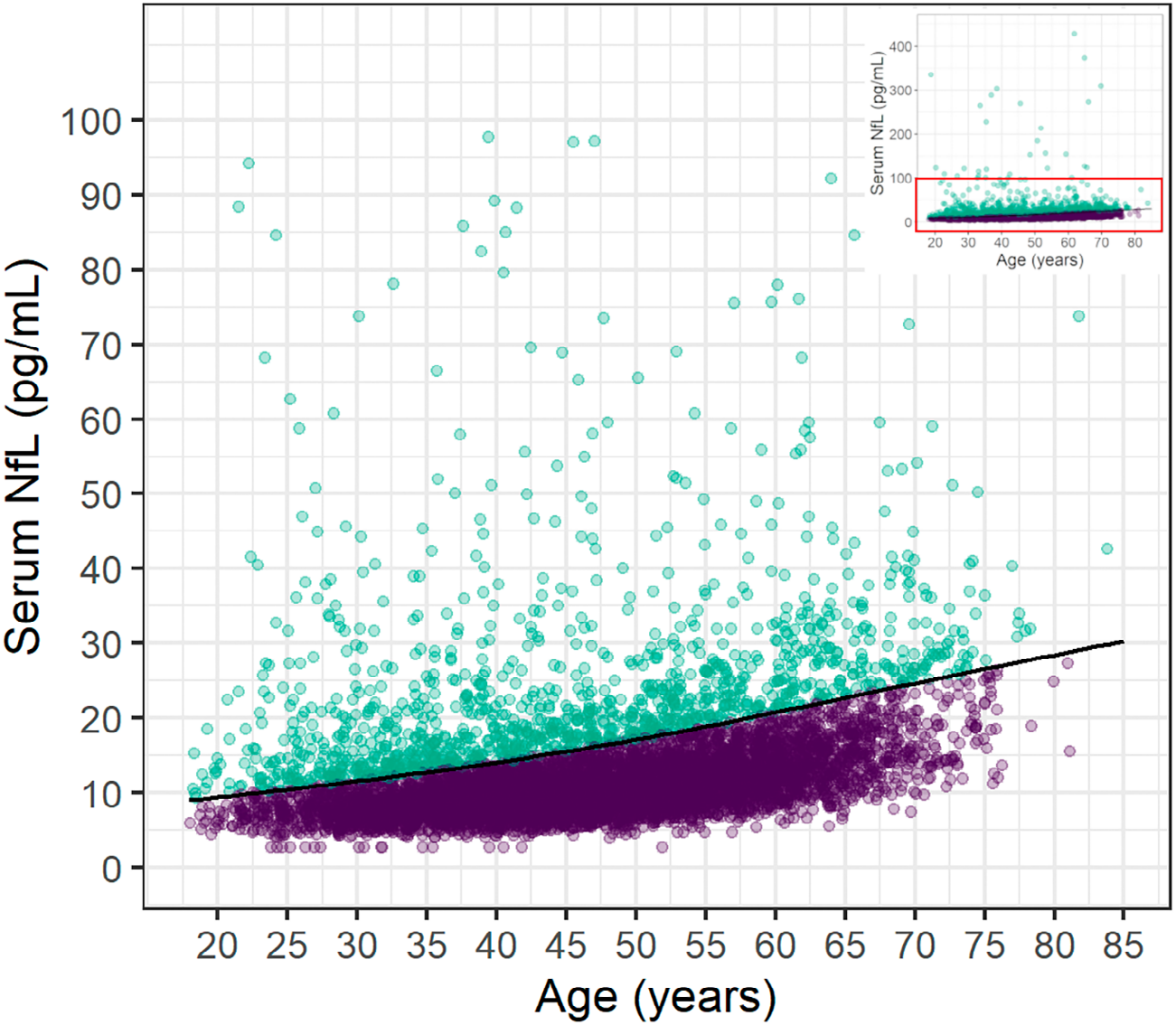
Serum neurofilament chain by age in the MS participants. Distribution of sNfL in the MS participants by age. The entire distribution is shown in the inset plot in the upper‐right corner, with the larger plot demonstrating the distribution in the range up to 100 pg/mL (red box in the inset plot). The overlaid line corresponds to the age‐normative 97.5th percentile curve derived from the healthy controls, with sNfL levels below the line (purple) and above the line (green) being categorized as “normal” (sNfL‐N) and “elevated” (sNfL‐E), respectively.

**Figure 3:**
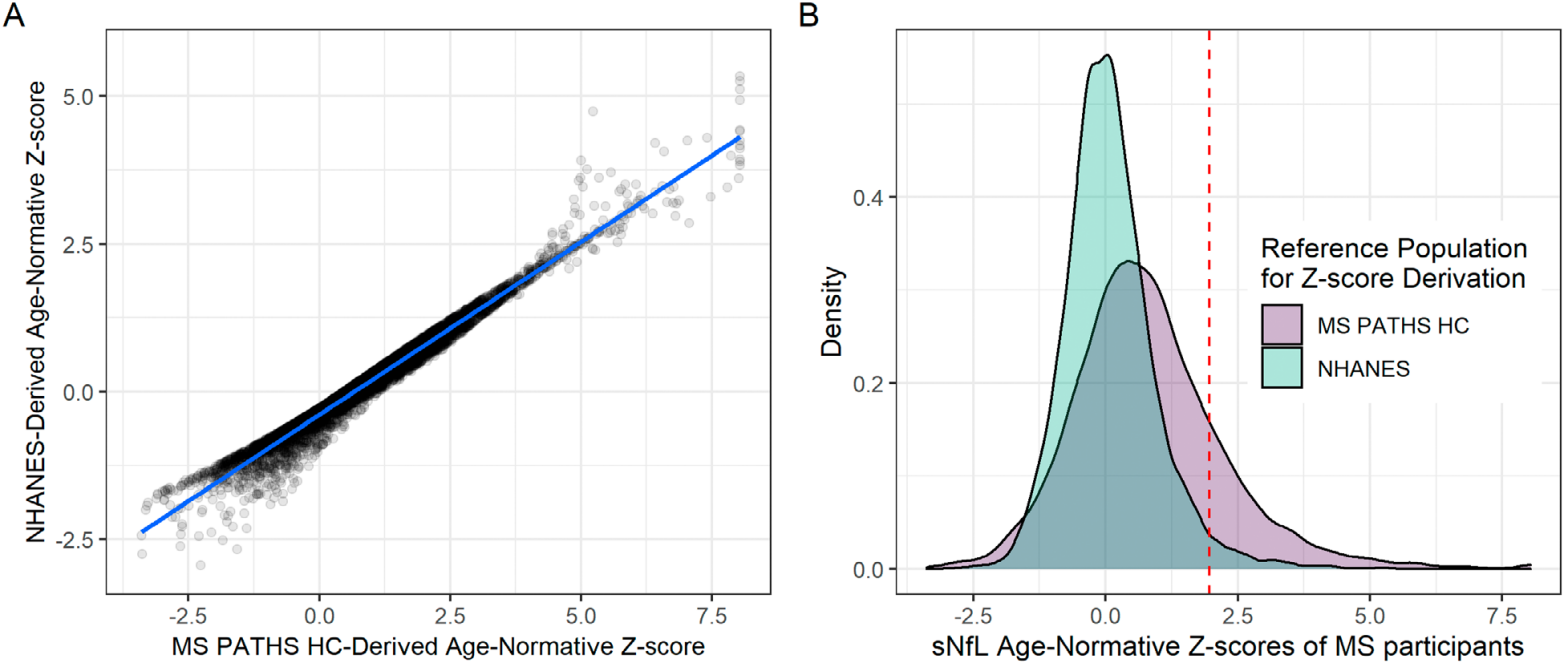
Associations between age‐normative Z‐scores derived using the MS PATHS HC vs NHANES. Panel A depicts the correlation of the MS participant age‐normative sNfL Z‐scores derived using the MS PATHS HC sNfL data (x‐axis) versus age‐normative Z‐scores derived using the NHANES reference population (y‐axis). Panel B depicts the distribution of the age‐normative sNfL Z‐scores in the MS population, normalized for age using MS PATHS HC (purple) or NHANES (green). Notably, while it is clear that the distribution of the MS PATHS HC‐derived Z‐scores deviate compared to the reference population (standardized to have a mean of zero and standard deviation of one in the MS PATHS HC), the distribution of the NHANES‐derived Z‐scores is similar to that of the reference population (i.e., the MS participants in MS PATHS do not appear to have abnormally elevated sNfL Z‐scores compared to the NHANES reference population).

As expected, sNfL levels increased with age in the HC (Fig. S1). Elevated sNfL levels above the age‐normative 97.5th percentile (derived from the HC) were present at baseline in 1238 MS participants (17.8%; Fig. 2 and Table 1). Interestingly, while the NHANES‐derived age‐normative sNfL Z‐scores for the MS participants demonstrated excellent correlations with those derived using the MS PATHS HC data (Fig. 3A), their distribution was similar to the NHANES reference population (Fig. 3B) and only 199 MS participants (2.9%) had sNfL levels above the NHANES‐derived age‐normative 97.5th percentile.

Initial analyses assessed the association of demographic characteristics, clinical characteristics, and comorbidities with the presence of elevated sNfL in MS (Table 2). Factors associated with increased odds of elevated sNfL included male sex (adjusted OR: 1.20, 95% CI: 1.04 to 1.39, *P* = 0.012), progressive disease subtype (adjusted OR: 1.63, 1.41 to 1.88, *P* < 0.001), diabetes mellitus (adjusted OR: 1.67, 95% CI: 1.31 to 2.12, *P* < 0.001), current smoking (adjusted OR: 1.21, 95% CI: 1.02 to 1.43, *P* = 0.029), and moderately to severely decreased eGFR (adjusted OR 5.21; 95% CI: 3.39 to 8.0, *P* < 0.001). Overall, higher age was associated with lower odds of elevated sNfL compared to younger patients (reference age group: 18–38 years). Individuals with short (<5 years) or long (>23 years) disease duration had higher odds of elevated sNfL compared to other disease duration categories. Increasing BMI was associated with lower odds of elevated sNfL, and this association was consistent across BMI categories. Use of an MS DMT was associated with lower odds of elevated sNfL, although 70% of patients with elevated sNfL were on an MS DMT. Overall, these findings were consistent in analyses of the continuous age‐normative sNfL Z‐scores, derived either from the MS PATHS HC (Table S3) or NHANES (Table S4).

**Table 2:** Associations of demographic and clinical characteristics with elevated sNfL.

Characteristics	Univariable analysis			Multivariable analysis		
	Odds ratio	95% CI	*P*‐value	Odds ratio	95% CI	*P*‐value
Age, years						
Q1: 18–36	—	—		—	—	
Q2: 37–42	**0.47**	**0.39, 0.57**	**<0.001**	**0.45**	**0.37, 0.55**	**<0.001**
Q3: 43–50	**0.38**	**0.31, 0.46**	**<0.001**	**0.34**	**0.27, 0.42**	**<0.001**
Q4: 51–58	**0.44**	**0.36, 0.53**	**<0.001**	**0.34**	**0.27, 0.43**	**<0.001**
Q5: >58	**0.66**	**0.55, 0.78**	**<0.001**	**0.39**	**0.31, 0.49**	**<0.001**
Sex						
Female	—	—		—	—	
Male	**1.11**	**0.97, 1.27**	**0.12**	**1.20**	**1.04, 1.39**	**0.012**
Body mass index, (kg/m^2^)						
18.5–24.9	—	—		—	—	
<18.5	**1.82**	**1.18, 2.76**	**0.006**	**1.84**	**1.16, 2.87**	**0.008**
25–29.9	**0.76**	**0.65, 0.90**	**<0.001**	**0.68**	**0.58, 0.81**	**<0.001**
30–39.9	**0.81**	**0.69, 0.95**	**0.009**	**0.65**	**0.55, 0.77**	**<0.001**
>40	0.85	0.64, 1.10	0.2	**0.61**	**0.45, 0.81**	**<0.001**
Disease duration, (years)						
Q1: < 5	—	—		—	—	
Q2: 5–9	**0.67**	**0.55, 0.82**	**<0.001**	**0.76**	**0.62, 0.94**	**0.012**
Q3: 10–15	**0.63**	**0.52, 0.78**	**<0.001**	**0.77**	**0.62, 0.96**	**0.020**
Q4: 16–23	**0.59**	**0.48, 0.72**	**<0.001**	**0.79**	**0.63, 0.99**	**0.045**
Q5 > 23	**0.82**	**0.68, 1.00**	**0.045**	1.02	0.80, 1.28	0.9
MS subtype						
CIS/RRMS	—	—		—	—	
Progressive MS	**1.62**	**1.42, 1.84**	**<0.001**	**1.63**	**1.41, 1.88**	**<0.001**
DMT class						
None	—	—		—	—	
IFN‐b/GA	**0.59**	**0.48, 0.72**	**<0.001**	**0.63**	**0.50, 0.78**	**<0.001**
Infusion/IRT	**0.80**	**0.66, 0.97**	**0.021**	**0.63**	**0.51, 0.77**	**<0.001**
Oral	**0.47**	**0.39, 0.57**	**<0.001**	**0.45**	**0.37, 0.55**	**<0.001**
Other or unknown	**0.75**	**0.59, 0.94**	**0.016**	0.78	0.61, 1.01	0.060
eGFR, (mL/min/1.73 m^2^)						
>90	—	—		—	—	
60–89	1.02	0.86, 1.21	0.8	1.20	1.00, 1.44	0.053
<60	**4.49**	**2.99, 6.73**	**<0.001**	**5.21**	**3.39, 8.00**	**<0.001**
Diabetes mellitus	**1.46**	**1.16, 1.83**	**<0.001**	**1.67**	**1.31, 2.12**	**<0.001**
Current smoking	1.12	0.96, 1.32	0.15	**1.21**	**1.02, 1.43**	**0.029**

Odds ratios were derived from logistic regression models assessing sNfL‐E versus sNfL‐N as the dependent variable. The multivariable model included all the characteristics listed in the table as independent variables. Statistically significant results (*P* < 0.05) are bolded.

sNfL‐E, elevated sNfL; sNfL‐N, “normal” sNfL; SD, standard deviation; IQR, interquartile range; CIS, clinically isolated syndrome; RRMS, relapsing–remitting MS; PDDS, Patient Determined Disease Steps; DMT, disease‐modifying therapy; IFN‐beta: interferon‐beta; GA, glatiramer acetate; IRT, immune reconstitution therapy; eGFR, estimated glomerular filtration rate.

### Baseline associations of sNfL with clinical disability and radiological measures

Cross‐sectional associations of sNfL group or MSPATHS HC‐derived age‐normative sNfL Z‐scores with neuroperformance measures and MRI volumetrics are shown in Table 3. Clinical disability was worse in those with sNfL‐E compared to those with sNfL‐N, as evidenced by higher self‐reported disability (adjusted OR—moderate vs mild disability: 1.39, 95% CI: 1.15 to 1.67, *P* < 0.001; severe vs mild disability: 2.26, 95% CI: 1.85 to 2.75, *P* < 0.001) and worse neuroperformance (adjusted difference in Z‐scores—walking speed: −0.54, 95% CI: −0.80 to −0.28; manual dexterity: −0.45, 95% CI −0.58 to −0.33; processing speed: −0.30, 95% CI: −0.38 to −0.22; *P* < 0.001 for all).

**Table 3:** Baseline associations of sNfL with neuroperformance measures and MRI volumetrics.

	sNfL‐E versus sNfL‐N			sNfL age normative Z‐score		
	Adjusted mean difference^1^	95% CI	*P*‐value	Adjusted beta coefficient^1^ ^,^ ^3^	95% CI	*P*‐value
Walking speed^2^	−0.54	−0.80 to −0.28	<0.001	−0.23	−0.30 to −0.16	<0.001
Manual dexterity^2^	−0.45	−0.58 to −0.33	<0.001	−0.14	−0.17 to −0.11	<0.001
Processing speed^2^	−0.30	−0.38 to −0.22	<0.001	−0.10	−0.12 to −0.08	<0.001
Brain parenchymal fraction^2^ ^,^ ^4^	−0.20	−0.28 to −0.12	<0.001	−0.06	−0.08 to −0.04	<0.001
T2 lesion volume^2^ ^,^ ^4^	0.42	0.33 to 0.51	<0.001	0.12	0.10 to 0.14	<0.001

^1^ Results derived from linear regression models adjusted for age, sex, BMI, disease duration, disease subtype, DMT class, eGFR, diabetes mellitus, and smoking status.

^2^ Neuroperformance domains were transformed to Z‐scores using regression‐based equations derived from a study of adult healthy volunteers. T2 lesion volume (normalized to total brain volume and log‐transformed) and BPF were converted to Z‐scores based on the distribution in the MS population.

^3^ Adjusted beta coefficients correspond to the change in the Z‐score for a given measure for a one‐unit increment in the sNfL age‐normative Z‐score (derived from the MS PATHS healthy control cohort). There was no evidence for deviation from linearity for any of the examined measures (likelihood ratio test for comparison between natural cubic spline model and linear model, *P*‐value >0.05 for all).

^4^ Brain parenchymal fraction and T2 lesion volume available for *n* = 3514 with MRI within 1 year of blood sampling (median [IQR] absolute time between blood sampling and MRI was 96 days [23 to 183 days]).

**Figure 4:**
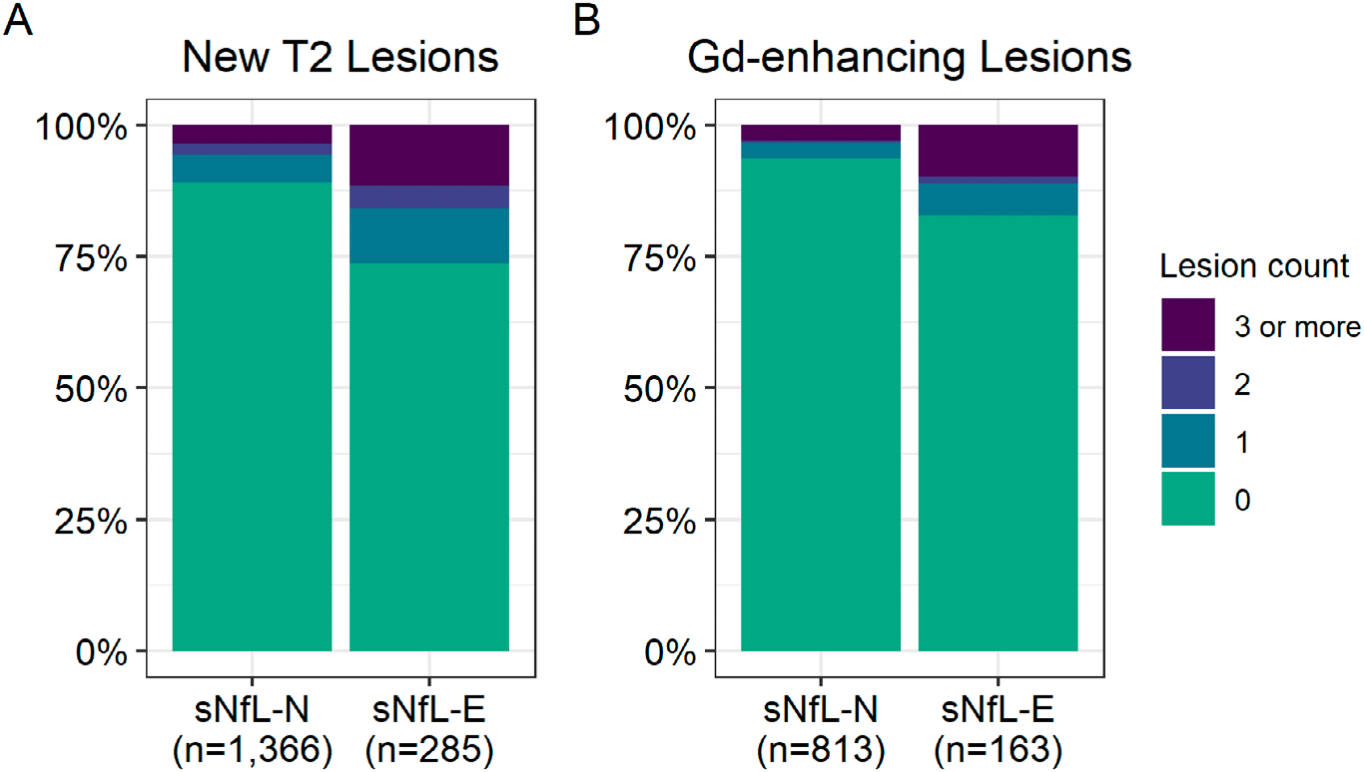
Serum neurofilament light chain group and associations with recent inflammatory disease activity. (A) New T2 lesion count compared to previous available brain MRI in sNfL‐N (“normal” sNfL) and sNfL‐E (“elevated” sNfL) MS participants. (B) Gadolinium (Gd)‐enhancing lesion count in sNfL‐N (“normal” sNfL) and sNfL‐E (“elevated” sNfL) MS participants with available contrast‐enhanced brain MRI within 60 days prior to sNfL measurement.

Furthermore, in those with available brain MRI within ±1 year of blood sampling (*n* = 3514; median absolute time between blood sampling and MRI: 96 days, IQR: 23 to 183 days), sNfL‐E participants had lower BPF and higher T2 lesion volume compared to sNfL‐N (adjusted differences in Z‐scores—BPF: −0.20, 95% CI: −0.28 to −0.12; T2 lesion volume: 0.42, 95% CI: 0.33 to 0.51; *P* < 0.001 for both). Findings were similar for the continuous MS PATHS HC‐derived age‐normative sNfL Z‐scores. Prior MRIs obtained at least 6 months before but within 2 years (median time from prior MRI: 1.0 years, IQR 0.8 to 1.3 years) were available for 1651 participants (*n* = 285 sNfL‐E, 17.3%) allowing assessment for interim radiological inflammatory disease activity (i.e., new T2 lesions). One or more new T2 lesions were found in 26.3% of sNfL‐E vs 10.9% of sNfL‐N participants (adjusted OR: 2.66, 95% CI: 1.86 to 3.77, *P* < 0.001; Fig. 4A), and consistent findings were found when assessing the MS PATHS HC‐derived sNfL Z‐score as a continuous variable (adjusted OR per one‐unit increase: 1.36; 95% CI: 1.23 to 1.50, *P* < 0.001).

Contrast‐enhanced MRI was available within 60 days prior to blood sampling for 976 participants (*n* = 163 sNfL‐E, 16.7%). One or more gadolinium‐enhancing lesions was detected in 17.2% of sNfL‐E vs 6.4% of sNfL‐N participants (adjusted OR: 3.68, 95% CI: 1.97 to 6.79, *P* < 0.001; Fig. 4B), and similar results were found when assessing the MS PATHS HC‐derived sNfL Z‐score as a continuous variable (adjusted OR: 1.50 per one‐unit increase, 95% CI: 1.25 to 1.79, *P* < 0.001). Of those with one or more gadolinium‐enhancing lesions (*n* = 80), 35% were sNfL‐E, whereas of those without gadolinium‐enhancing lesions (*n* = 896) 6.4% were sNfL‐E.

Baseline associations of sNfL with clinical disability and radiological measures were also assessed utilizing NHANES‐derived age‐normative Z‐scores and exhibited similar relationships (Table S5).

### Longitudinal analysis of brain atrophy and new T2 lesions

We examined associations between sNfL levels, new T2 lesion development, and whole brain atrophy over a follow‐up period of up to 2 years, in 2251 MS participants (*n* = 400 sNfL‐E, 17.8%) with at least 6 months of available MRI follow‐up (median follow‐up: 1.7 years; IQR 1.1 to 1.9 years). Longitudinal MRI was also available for 148 HC participants (median follow‐up: 1.2 years, IQR: 1.0 to 2.1 years).

**Figure 5:**
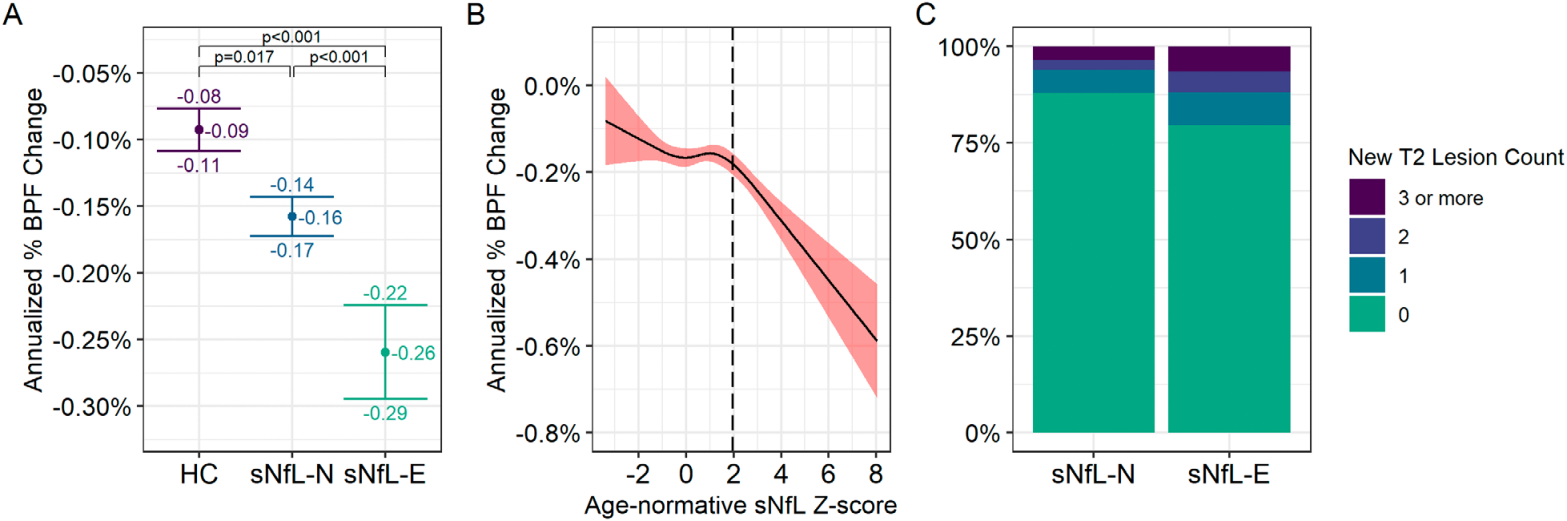
Associations of serum neurofilament light chain with prospective brain atrophy and T2 lesion development. (A) Comparison of annualized percent change (mean and 95% confidence intervals) in brain parenchymal fraction between HC (healthy controls), sNfL‐N (“normal” sNfL), and sNfL‐E (“elevated” sNfL) MS participants. (B) Relationship of annualized percent change in brain parenchymal fraction (BPF) with age‐normative sNfL Z‐scores (derived from MS PATHS HC) in the MS participants. The estimates were derived from a mixed effects regression model using restricted cubic splines to model the sNfL Z‐score (likelihood ratio test compared to linear model *P* = 0.002). The solid line corresponds to the estimated mean annualized percent change in BPF, and the bounds of the shaded area to the 95% confidence intervals. The vertical dashed line corresponds to the 97.5th percentile cut‐off used to define sNfL‐E versus sNfL‐N (i.e., age‐adjusted sNfL Z‐score of 1.96). (C) New T2 lesion count during follow‐up in in sNfL‐N (“normal” sNfL) and sNfL‐E (“elevated” sNfL) MS participants.

sNfL‐E participants exhibited 63% faster whole brain atrophy compared to sNfL‐N participants (annualized percent change in BPF: −0.26%/year vs −0.16%/year; adjusted difference: −0.10%/year, 95% CI: −0.14% to −0.06%, *P* < 0.001; Fig. 5A). Both groups exhibited faster rates of brain atrophy compared to HC (annualized percent change in BPF in HC −0.09%/year; adjusted differences vs HC—sNfL‐E: −0.16%/year, 95% CI: −0.20% to −0.12%, *P* < 0.001; sNfL‐N: −0.06%/year, 95% CI: −0.09% to −0.04%, *P* = 0.017). Higher MS PATHS HC‐derived age‐normative sNfL Z‐scores were associated with faster brain atrophy (adjusted change in annualized percent brain atrophy: −0.026%/year per one‐unit increase, 95% CI: −0.03% to −0.02%, *P* < 0.001), but this relationship appeared non‐linear (likelihood ratio test *P* = 0.003), with relatively stable brain atrophy rates for sNfL Z‐scores less than two (i.e., corresponding to the 97.5^th^ percentile [z = 1.96] cut‐off used to identify sNfL‐E) but progressively increasing rates of brain volume loss for those with higher sNfL Z‐scores (Fig. 5B). One or more new T2 lesions was detected during follow‐up in 20.5% of sNfL‐E vs 12.1% of sNfL‐N participants (adjusted OR: 1.94, 95% CI: 1.42 to 2.62, *P* < 0.001). Findings were consistent when assessing the MS PATHS HC‐derived age‐normative sNfL Z‐score as a continuous variable (adjusted OR: 1.19 per one‐unit increase, 95% CI: 1.10 to 1.29, *P* < 0.001).

These analyses were also performed utilizing NHANES‐derived age‐normative Z‐scores with similar findings for brain atrophy (adjusted change in annualized percent brain atrophy: −0.041%/year per one‐unit increase, 95% CI: −0.050% to −0.033%, *P* < 0.001; Fig. S2) and new T2 lesion development (adjusted OR: 1.34 per one‐unit increase, 95% CI: 1.16 to 1.54, *P* < 0.001).

## Discussion

In summary, we assessed sNfL, measured using a novel high‐throughput immunoassay on an existing clinical laboratory platform, in a large, multi‐center, real‐world population of PwMS. We found that a significant proportion of PwMS had elevated sNfL relative to age‐adjusted reference ranges derived from HC. Factors associated with elevated sNfL for age in MS included demographic factors (male sex, younger age), clinical characteristics (progressive disease subtype, lack of DMT use, short or long disease duration), and comorbidities (impaired renal function, diabetes mellitus, and smoking). Notably, higher BMI was associated with lower sNfL. Furthermore, sNfL elevations were associated with more severe physical and cognitive disability, brain atrophy, and radiological inflammatory disease activity.

Previous studies have established a strong association between age and sNfL levels.^3^ Notably, we found that, when accounting for the age‐adjusted distribution of sNfL in the MS PATHS HC, younger PwMS were more likely to have elevated sNfL levels for age. This is consistent with the observation that overt inflammatory disease activity (and consequent neuro‐axonal injury) is more frequent in younger PwMS and decreases with aging.^33^ Furthermore, shorter disease duration (<5 years) was also independently associated with elevated sNfL, consistent with this phenomenon, although those with longer disease duration (> 3 years) also exhibited similarly elevated sNfL, which could relate to increasing contribution of neurodegenerative processes. Furthermore, male sex was found to be associated with higher age‐adjusted sNfL. Natural history studies have suggested that among people with MS, males are at a higher risk of disability and progressive disease, and it is likely that this finding is reflective of this association. However, while an individual participant‐level meta‐analysis of CSF NfL also found higher levels in males with MS, this was also observed in healthy controls and other non‐inflammatory neurological diseases.^34^ This observation needs to be explored further in large control populations in order to assess the contribution of biological sex to blood NfL levels independent of neurological disease.

Additionally, our findings support the importance of accounting for comorbidities when interpreting sNfL. Our observation of a relationship between reduced eGFR and higher sNfL is consistent with recent reports of large reference populations (including NHANES), as well as smaller prior reports of older adults without neurological disease.^9^, ^10^, ^12^, ^13^ The precise mechanism by which renal function is associated with sNfL is not clear but could be related to possible renal clearance of blood NfL and/or neurological complications of renal disease.^35^ This is especially relevant in people with progressive MS, who may have lower than predicted eGFR.^36^ Diabetes mellitus and smoking were also associated with higher sNfL, which could be related to complications such as diabetic neuropathy and cerebrovascular disease, but these factors may also be associated with a pro‐inflammatory state and exacerbate underlying MS disease processes.^37^, ^38^ BMI, however, exhibited an inverse association with sNfL, consistent with prior reports, possibly related to a dilutional effect of blood volume which is highly correlated with BMI.^9^, ^11^, ^12^, ^13^

We also confirmed that elevated sNfL was associated cross‐sectionally with both physical and cognitive disability in MS, as well as lower BPF and higher T2 lesion volume. These associations were generally modest, in line with prior smaller studies. This is consistent with the perception of sNfL as a dynamic biomarker reflecting recent and ongoing neuro‐axonal injury, whereas established clinical disability and brain atrophy in MS represent the cumulative effects of preceding neuro‐axonal injury.^5^, ^6^ Furthermore, elevated sNfL was associated with markedly accelerated short‐term brain atrophy, and significantly higher odds of prior, ongoing and future inflammatory disease activity. Importantly though, rates of whole brain atrophy were also faster compared to HC for those with sNfL within the age‐normative reference range.

Interestingly, when using NHANES as the reference population, age‐normative sNfL levels in the MS participants were not elevated compared to the reference distribution, whereas when using the MS PATHS HC as the reference population, we were clearly able to detect sNfL elevations in the MS participants. Notably, NHANES‐derived and MS PATHS HC‐derived age‐normative sNfL Z‐scores exhibited excellent correlations and this seemed to represent a systematic difference between the two reference populations. This observation is unlikely to be related to a technical issue due to different collection procedures and/or processing, given that sNfL levels have been previously reported to remain stable in unprocessed blood samples stored up to 7 days at room temperature, to withstand repeat freeze–thaw cycles, and to be similar across different serum collection tube types.^20^, ^39^, ^40^ Given the above, we speculate that this difference may relate to selection bias of individuals recruited from tertiary referral centers (with the MS PATHS HC sampled from a similar population), whereas NHANES is designed to be a representative sample of the non‐institutionalized US population.^41^ This highlights the importance of considering a reference population that is representative of the population under consideration when interpreting sNfL levels at an individual level. Important strengths of our study include the large sample size with standardized MRIs available for a large subset of the cohort, multi‐center data from across two continents, the incorporation of standardized collection of measures assessing ambulatory disability, manual dexterity and cognitive disability, and the availability of data related to comorbidities. Furthermore, we employed a high‐throughput, scalable immunoassay for sNfL measurement that can be run on an existing routine clinical laboratory platform, with measurements demonstrating good correlation with Simoa, and consistent associations with clinical and radiological measures.

Our study bears several limitations that warrant discussion. sNfL was measured only at a single timepoint for this analysis. Given the dynamic nature of inflammation and consequent neuro‐axonal injury in MS, it is likely that serial measurements of sNfL will be more informative.^7^ Serial blood sampling is being performed as part of MS PATHS and future planned analyses will investigate the utility of serial sNfL measurements. Furthermore, we were unable to perform a detailed analysis of the impact of inflammatory disease activity at the time of sampling on sNfL and its relationship with outcomes, given that exact timing of relapses and contrast‐enhanced MRI was not available routinely for the studied population within close proximity to blood sampling. Additionally, the duration of follow‐up at present in MS PATHS was insufficient to assess for clinical disability progression in a sufficiently large sample of participants. Longitudinal follow‐up of MS PATHS study participants is ongoing, and this is an important outcome of interest that will be assessed. Furthermore, the sample size of the MS PATHS HC population was relatively small, but, as we observed, the use of an unrelated reference population (i.e., NHANES) may not have been appropriate for our MS population, precluding the ability to detect abnormal sNfL elevations, although analyses of continuous age‐normative sNfL Z‐scores demonstrated consistent findings. Finally, since data in MS PATHS are collected as part of routine clinical care, measures including MRI and creatinine/eGFR were not available for the entire cohort.

In conclusion, our study supports that sNfL, measured using a high‐throughput and scalable assay, is associated with clinical disability, inflammatory disease activity, and whole brain atrophy in MS, but interpretation needs to account for body mass index and comorbidities including impaired renal function, diabetes, and smoking. Longitudinal follow‐up of MS PATHS study participants is ongoing, and planned future work includes examining associations of sNfL (including serial sNfL measurements) with disability progression and long‐term radiological outcomes.

## Author Contributions

E.S.S., K.C.F., E.M.M., and P.A.C. contributed to the conception and design of the study. E.S.S., K.C.F., C.M.M., M.D.S., M.R., C.M.H., M.H.H., R.C., S.B.S., G.A., X.M., M.C., R.T.N., M.Q., L.B.K., J.A.N., K.A., T.Z., R.R., E.F., R.A.B., E.M.M., and P.A.C. contributed to the acquisition and/or analysis of the data. E.S.S., K.C.F., E.M.M., and P.A.C. drafted the text and prepared the figures.

## Conflict of Interest

Elias Sotirchos reports: scientific advisory board and/or consulting for Alexion, Viela Bio, Horizon Therapeutics, Genentech, and Ad Scientiam; speaking honoraria from Alexion, Viela Bio, and Biogen. Kathryn Fitzgerald, Matthew Smith, Maria Reyes‐Mantilla, Ryan Canissario, Min Qiao, and Sarah Simmons have nothing to disclose. Carol Singh and Elizabeth Fisher are employees of Biogen and hold stock/stock options in the company. Carrie Hersh reports: scientific advisory board and/or consulting for Biogen, Novartis, Genentech, Genzyme, EMD Serono, TG Therapeutics, and Bristol‐Myers Squibb; compensation for serving on speakers bureaus for Genzyme and Biogen; research support from Biogen, Novartis, and Genentech. Megan Hyland reports: research support from Biogen. Georgina Arrambide reports: consulting services or participation in advisory boards from Sanofi, Merck and Roche; travel expenses for scientific meetings from Novartis, Roche, Stendhal and ECTRIMS; speaking honoraria from Sanofi, Roche, and Merck. Xavier Montalban reports: speaking honoraria and travel expenses for scientific meetings, has been a steering committee member of clinical trials or participated in advisory boards of clinical trials in the past 3 years with Actelion, Alexion, Biogen, Celgene, EMD Serono, Genzyme, Immunic, Medday, Merck, Mylan, Novartis, Roche, Sanofi‐Genzyme, and Teva Pharmaceutical. Manuel Comabella reports: compensation for consulting services and speaking honoraria from Bayer Schering Pharma, Merk Serono, Biogen‐Idec, Teva Pharmaceuticals, Sanofi‐Aventis, and Novartis. Robert Naismith reports: scientific advisory board and/or consulting for Abata Therapeutics, Banner Life Sciences, BeiGene, Biogen, Bristol Myers Squibb, Genentech, Genzyme, Janssen, GW Therapeutics, Horizon Therapeutics, Lundbeck, NervGen, TG Therapeutics. Lauren Krupp reports: scientific advisory board and/or consulting for Biogen, Novartis, Janssen, Gerson Lehrman, Sanofi, and Biogen; research support from Biogen. Jacqueline Nicholas reports: scientific advisory board and/or consulting for Novartis, Genentech, Greenwich Biosciences, Biogen, EMD Serono, and TG Therapeutics; compensation for serving on speakers bureaus for EMD Serono, Alexion, Viela Bio, and Bristol Myers Squibb; research support from Novartis, Biogen, and Genentech. Katja Akgün reports: scientific advisory board and/or consulting for Roche, Sanofi, Alexion, Teva, Biogen, and Celgene. Tjalf Ziemssen reports: scientific advisory board and/or consulting for Biogen, Roche, Novartis, Celgene, and Merck; compensation for serving on speakers bureaus for Roche, Novartis, Merck, Sanofi, Celgene, and Biogen; research support from Biogen, Novartis, Merck, and Sanofi. Richard Rudick is a former Biogen employee and holds stock in the company. Robert Bermel reports: scientific advisory board and/or consulting for Astra Zeneca, Biogen, EMD Serono, Genzyme, Genentech, Novartis, and VielaBio, research support from Biogen, Genentech, and Novartis, and shared rights to intellectual property underlying the Multiple Sclerosis Performance Test, currently licensed to Qr8 Health and Biogen. Ellen Mowry reports: research support from Biogen, Teva, and Genentech, and royalties for editorial duties from UpToDate. Peter Calabresi reports: consulting fees from Biogen, Nervgen, Idorsia, Avidea (now Vaccitech), and Disarm Therapeutics (now Lilly); research support from Principia and Genentech.

## Supporting information


**Table S1.** Characteristics of the healthy controls.
**Table S2.** Distribution of MS participants and healthy controls by MS PATHS site.
**Table S3.** Associations of demographic and clinical characteristics with age‐normative sNfL Z‐scores (derived using the sNfL distribution in the MS PATHS healthy control cohort).
**Table S4.** Associations of demographic and clinical characteristics with age‐normative sNfL Z‐scores (derived using the sNfL distribution in the NHANES reference population).
**Table S5.** Associations of MS participant age‐normative NHANES‐derived sNfL Z‐scores with neuroperformance measures, MRI volumetrics and new T2 lesion development.
**Figure S1.** Serum neurofilament light chain levels by age in the healthy control cohort.
**Figure S2.** Associations of age‐normative sNfL Z‐scores derived using NHANES with prospective whole brain atrophy in the MS participants.

## Data Availability

Requests for individual participant de‐identified data would be considered from qualified investigators, based on information provided, including the proposed use and analysis plan. Requests to access the datasets should be directed to datasharing@biogen.com. Proposals will be reviewed by the MS PATHS data use committee. If the proposed use is appropriate, a data sharing agreement will be put in place before a fully de‐identified version of the dataset, including the data dictionary, is made available.
