# Supplementary material for: Associations of sNfL with clinico‐radiological measures in a large MS population
Source: Ann Clin Transl Neurol. 2022 Nov 25;10(1):84–97. doi: 10.1002/acn3.51704 (PMC9852396; doi:10.1002/acn3.51704)
Supplement: Supplementary file 1 — Table S1. Characteristics of the healthy controls. Table S2. Distribution of MS participants and healthy controls by MS PATHS site. Table S3. Associations of demographic and clinical characteristics with age‐normative sNfL Z‐scores (derived using the sNfL distribution in the MS PATHS healthy control cohort). Table S4. Associations of demographic and clinical characteristics with age‐normative sNfL Z‐scores (derived using the sNfL distribution in the NHANES reference population). Table S5. Associations of MS participant age‐normative NHANES‐derived sNfL Z‐scores with neuroperformance measures, MRI volumetrics and new T2 lesion development. Figure S1. Serum neurofilament light chain levels by age in the healthy control cohort. Figure S2. Associations of age‐normative sNfL Z‐scores derived using NHANES with prospective whole brain atrophy in the MS participants. [file ACN3-10-84-s001.pdf]

Supplementary Table 1. Characteristics of the healthy controls.

	**N = 201**
Age (years), mean (SD)	42.6 (10.8)
Female, n (%)	149 (74%)
Race, n (%)	
White	169 (84%)
Black	14 (7.0%)
Other or Not reported	18 (9.0%)
Body Mass Index (kg/m^2), median (IQR)	26.6 (23.6, 30.3)
Diabetes Mellitus, n (%)	6 (3.0%)
Current smoker, n (%)	15 (7.5%)
Serum NfL (pg/mL), median (IQR)	8.50 (6.60, 10.80)
Brain Parenchymal Fraction, mean (SD)	0.869 (0.013)

Abbreviations – SD: standard deviation; IQR: inter-quartile range

Supplementary Table 2. Distribution of MS participants and healthy controls by MS PATHS site

**Site**	**MS (n=6,974)**	**Healthy Controls (n=201)**
1	301	14
2	27	-
3	471	40
4	297	-
5	2,277	51
6	396	-
7	1,370	41
8	633	49
9	416	-
10	786	6

Supplementary Table 3. Associations of demographic and clinical characteristics with age-normative sNfL Z-scores (derived using the sNfL distribution in the MS PATHS healthy control cohort).

	Univariate Analysis			Multivariable Analysis		
**Characteristic**	**Beta**	**95% CI**	**p-value**	**Beta**	**95% CI**	**p-value**
Age, years						
Q1: 18-36	—	—		—	—	
Q2: 37-42	**-0.41**	**-0.52, -0.30**	**<0.001**	**-0.41**	**-0.52, -0.31**	**<0.001**
Q3: 43-50	**-0.54**	**-0.65, -0.44**	**<0.001**	**-0.57**	**-0.68, -0.46**	**<0.001**
Q4: 51-58	**-0.43**	**-0.53, -0.32**	**<0.001**	**-0.55**	**-0.66, -0.44**	**<0.001**
Q5: >58	-0.09	-0.20, 0.01	0.092	**-0.40**	**-0.52, -0.28**	**<0.001**
Sex						
Female	—	—		—	—	
Male	**0.12**	**0.05, 0.20**	**0.001**	**0.16**	**0.08, 0.23**	**<0.001**
Body Mass Index, (kg/m^2)						
18.5-24.9	—	—		—	—	
<18.5	**0.44**	**0.16, 0.72**	**0.002**	**0.41**	**0.14, 0.68**	**0.003**
25-29.9	**-0.16**	**-0.25, -0.07**	**<0.001**	**-0.24**	**-0.33, -0.16**	**<0.001**
30-39.9	**-0.25**	**-0.34, -0.16**	**<0.001**	**-0.40**	**-0.49, -0.32**	**<0.001**
>40	**-0.38**	**-0.53, -0.23**	**<0.001**	**-0.57**	**-0.71, -0.42**	**<0.001**
Disease Duration, (years)						
Q1: < 5	—	—		—	—	
Q2: 5-9	**-0.24**	**-0.35, -0.12**	**<0.001**	**-0.16**	**-0.27, -0.05**	**0.003**
Q3: 10-15	**-0.21**	**-0.33, -0.10**	**<0.001**	**-0.13**	**-0.24, -0.02**	**0.021**
Q4: 16-23	**-0.19**	**-0.31, -0.08**	**<0.001**	-0.08	-0.19, 0.03	0.2
Q5 >23	-0.06	-0.17, 0.05	0.3	-0.05	-0.16, 0.07	0.5
MS Subtype						
CIS/RRMS	—	—		—	—	
Progressive MS	**0.35**	**0.28, 0.43**	**<0.001**	**0.29**	**0.21, 0.36**	**<0.001**
DMT Class						
None	—	—		—	—	
IFN-b/GA	**-0.29**	**-0.40, -0.17**	**<0.001**	**-0.24**	**-0.36, -0.13**	**<0.001**
Infusion/IRT	**-0.15**	**-0.26, -0.04**	**0.009**	**-0.27**	**-0.38, -0.16**	**<0.001**
Oral	**-0.61**	**-0.72, -0.50**	**<0.001**	**-0.60**	**-0.71, -0.49**	**<0.001**
Other or Unknown	**-0.16**	**-0.30, -0.02**	**0.024**	-0.09	-0.23, 0.04	0.2
eGFR, (mL/min/1.73m^2)						
>90	—	—		—	—	
60-89	**0.11**	**0.02, 0.21**	**0.018**	**0.16**	**0.07, 0.25**	**<0.001**
<60	**1.3**	**1.0, 1.6**	**<0.001**	**1.2**	**1.0, 1.5**	**<0.001**
Diabetes Mellitus	**0.22**	**0.08, 0.35**	**0.002**	**0.30**	**0.17, 0.44**	**<0.001**
Current Smoking	0.08	-0.01, 0.17	0.08	**0.14**	**0.05, 0.22**	**0.002**

Beta coefficients were derived from linear regression models including the sNfL Z-score as the dependent variable. The multivariable model included all the characteristics listed in the table as independent variables. Beta coefficients correspond to the change in the sNfL Z-score for the level of each categorical variable compared to the reference.

Abbreviations – sNfL-E: elevated sNfL; sNfL-N: "normal" sNfL; SD: standard deviation; IQR: inter-quartile range; CIS: clinically isolated syndrome; RRMS: relapsing-remitting MS; PDDS: Patient Determined Disease Steps; DMT: disease-modifying therapy; IFN-beta: interferon-beta; GA: glatiramer acetate; IRT: immune reconstitution therapy; eGFR: estimated glomerular filtration rate

Supplementary Table 4. Associations of demographic and clinical characteristics with age-normative sNfL Z-scores (derived using the sNfL distribution in the NHANES reference population).

	Univariate Analysis			Multivariable Analysis		
**Characteristic**	**Beta**	**95% CI**	**p-value**	**Beta**	**95% CI**	**p-value**
Age, years						
Q1: 18-36	—	—		—	—	
Q2: 37-42	**-0.13**	**-0.20, -0.07**	**<0.001**	**-0.14**	**-0.20, -0.07**	**<0.001**
Q3: 43-50	**-0.17**	**-0.24, -0.11**	**<0.001**	**-0.19**	**-0.25, -0.13**	**<0.001**
Q4: 51-58	**-0.15**	**-0.21, -0.08**	**<0.001**	**-0.22**	**-0.29, -0.15**	**<0.001**
Q5: >58	**-0.11**	**-0.18, -0.05**	**<0.001**	**-0.29**	**-0.36, -0.22**	**<0.001**
Sex						
Female	—	—		—	—	
Male	**0.06**	**0.02, 0.11**	**0.005**	**0.09**	**0.04, 0.13**	**<0.001**
Body Mass Index, (kg/m^2)						
18.5-24.9	—	—		—	—	
<18.5	**0.26**	**0.09, 0.43**	**0.003**	**0.24**	**0.08, 0.40**	**0.004**
25-29.9	**-0.09**	**-0.15, -0.04**	**<0.001**	**-0.14**	**-0.19, -0.09**	**<0.001**
30-39.9	**-0.15**	**-0.20, -0.09**	**<0.001**	**-0.24**	**-0.30, -0.19**	**<0.001**
>40	**-0.19**	**-0.28, -0.11**	**<0.001**	**-0.32**	**-0.40, -0.23**	**<0.001**
Disease Duration, (years)						
Q1: < 5	—	—		—	—	
Q2: 5-9	**-0.13**	**-0.20, -0.06**	**<0.001**	**-0.09**	**-0.15, -0.02**	**0.009**
Q3: 10-15	**-0.10**	**-0.17, -0.03**	**0.003**	**-0.06**	**-0.13, 0.01**	**0.07**
Q4: 16-23	**-0.09**	**-0.16, -0.02**	**0.008**	-0.04	-0.11, 0.03	0.3
Q5 >23	-0.05	-0.12, 0.01	0.11	-0.02	-0.09, 0.05	0.5
MS Subtype						
CIS/RRMS	—	—		—	—	
Progressive MS	**0.20**	**0.15, 0.24**	**<0.001**	**0.17**	**0.12, 0.21**	**<0.001**
DMT Class						
None	—	—		—	—	
IFN-b/GA	**-0.14**	**-0.21, -0.07**	**<0.001**	**-0.13**	**-0.20, -0.06**	**<0.001**
Infusion/IRT	-0.05	-0.12, 0.02	0.2	**-0.15**	**-0.22, -0.08**	**<0.001**
Oral	**-0.31**	**-0.37, -0.24**	**<0.001**	**-0.34**	**-0.40, -0.27**	**<0.001**
Other or Unknown	-0.09	-0.17, -0.01	0.034	-0.06	-0.14, 0.02	0.2
eGFR, (mL/min/1.73m^2)						
>90	—	—		—	—	
60-89	0.05	-0.01, 0.11	0.059	**0.09**	**0.04, 0.15**	**<0.001**
<60	**0.70**	**0.52, 0.87**	**<0.001**	**0.73**	**0.56, 0.89**	**<0.001**
Diabetes Mellitus	**0.12**	**0.03, 0.20**	**0.005**	**0.18**	**0.10, 0.26**	**<0.001**
Current Smoking	**0.06**	**0.01, 0.11**	**0.032**	**0.08**	**0.03, 0.14**	**0.002**

Beta coefficients were derived from linear regression models including the sNfL Z-score as the dependent variable. The multivariable model included all the characteristics listed in the table as independent variables. Beta coefficients correspond to the change in the sNfL Z-score for the level of each categorical variable compared to the reference.

Abbreviations – sNfL-E: elevated sNfL; sNfL-N: "normal" sNfL; SD: standard deviation; IQR: inter-quartile range; CIS: clinically isolated syndrome; RRMS: relapsing-remitting MS; PDDS: Patient Determined Disease Steps; DMT: disease-modifying therapy; IFN-beta: interferon-beta; GA: glatiramer acetate; IRT: immune reconstitution therapy; eGFR: estimated glomerular filtration rate

Supplementary Table 5. Associations of MS participant age-normative NHANES-derived sNfL Z-scores with neuroperformance measures, MRI volumetrics and new T2 lesion development.

	**sNfL age normative Z-score**		
	**Adjusted Beta Coefficient[1,3]**	**95% CI**	**p-value**
Walking speed[2]	**-0.38**	**-0.49 to -0.26**	**<0.001**
Manual dexterity[2]	**-0.23**	**-0.29 to -0.17**	**<0.001**
Processing speed[2]	**-0.17**	**-0.21 to -0.13**	**<0.001**
Brain parenchymal fraction[2,4]	**-0.09**	**-0.12 to -0.05**	**<0.001**
T2 lesion volume[2,4]	**0.19**	**0.16 to 0.23**	**<0.001**
	**Adjusted Odds Ratio[5]**	**95% CI**	**p-value**
New T2 lesion compared to prior MRI scan (retrospective)[6]	**1.63**	**1.38 to 1.93**	**<0.001**
Presence of Gd-enhancing lesion[6]	**2.00**	**1.49 to 2.68**	**<0.001**

[1]Results derived from linear regression models adjusted for age, sex, BMI, disease duration, disease subtype, DMT class, eGFR, diabetes mellitus and smoking status.

[2]Neuroperformance domains were transformed to Z-scores using regression-based equations derived from a study of adult healthy volunteers. T2 lesion volume (normalized to total brain volume and log-transformed) and BPF were converted to Z-scores based on the distribution in the MS population.

[3]Adjusted beta coefficients correspond to the change in the Z-score for a given measure for a one-unit increment in the sNfL age-normative Z-score (derived from the NHANES reference population). There was no evidence for deviation from linearity for any of the examined measures (likelihood ratio test for comparison between natural cubic spline model and linear model, p-value > 0.05 for all).

[4]Brain parenchymal fraction and T2 lesion volume available for n=3,514 with MRI within 1 year of blood sampling (median [IQR] absolute time between blood sampling and MRI was 96 days [23 to 183 days]).

[5]Results derived from logistic regression models adjusted for age, sex, BMI, disease duration, disease subtype, DMT class, eGFR, diabetes mellitus and smoking status. The adjusted odds ratios are per one-unit increment in the sNfL age-normative Z-score (derived from the NHANES reference population).

[6]Prior MRIs obtained at least 6 months before but within 2 years available for n=1,651 MS participants and contrast-enhanced MRI available within 60 days prior to blood sampling for n=976 participants.

Supplementary Figure 1. Serum neurofilament light chain levels by age in the healthy control cohort.

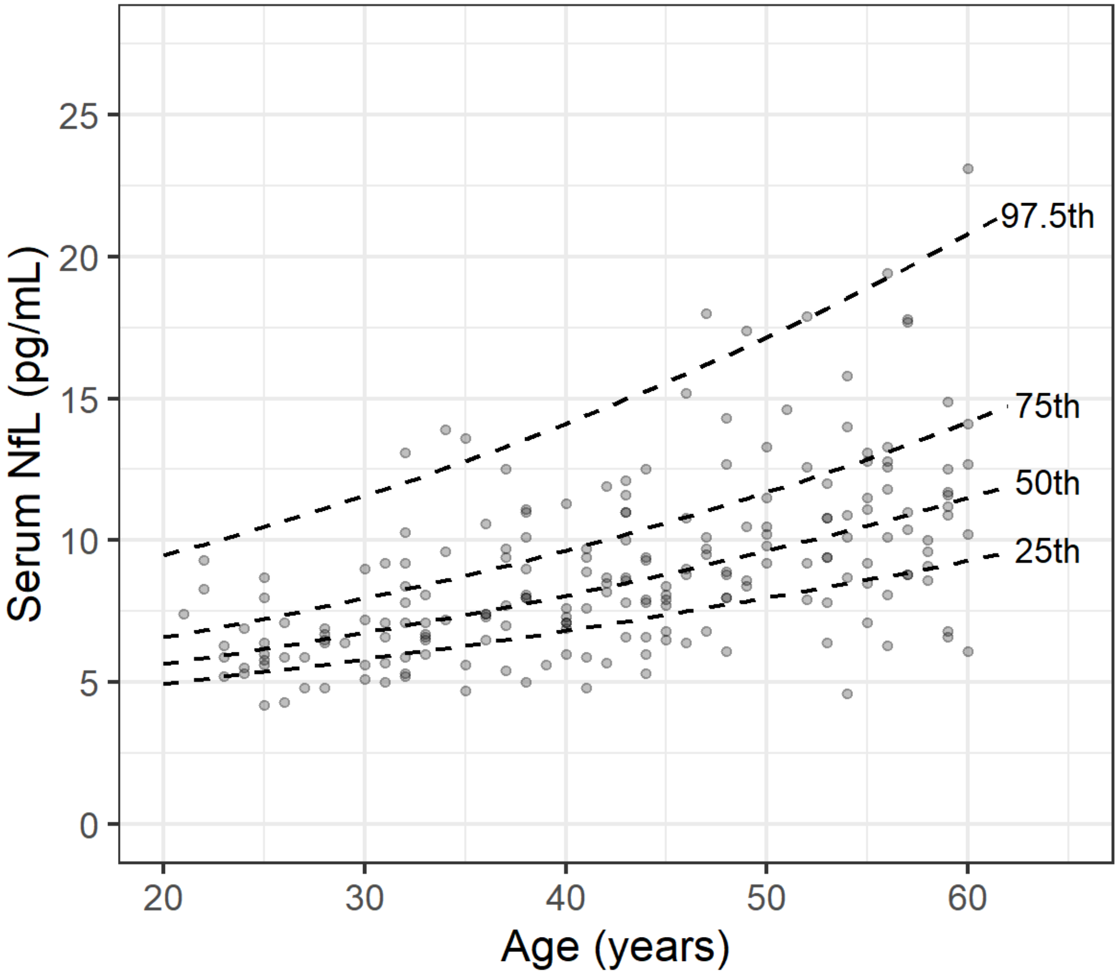

Association of serum neurofilament chain with age in the healthy controls (n=201). The overlaid dashed line corresponds to the age-normative 97.5th percentile curve.

Supplementary Figure 2. Associations of age-normative sNfL Z-scores derived using NHANES with prospective whole brain atrophy in the MS participants.

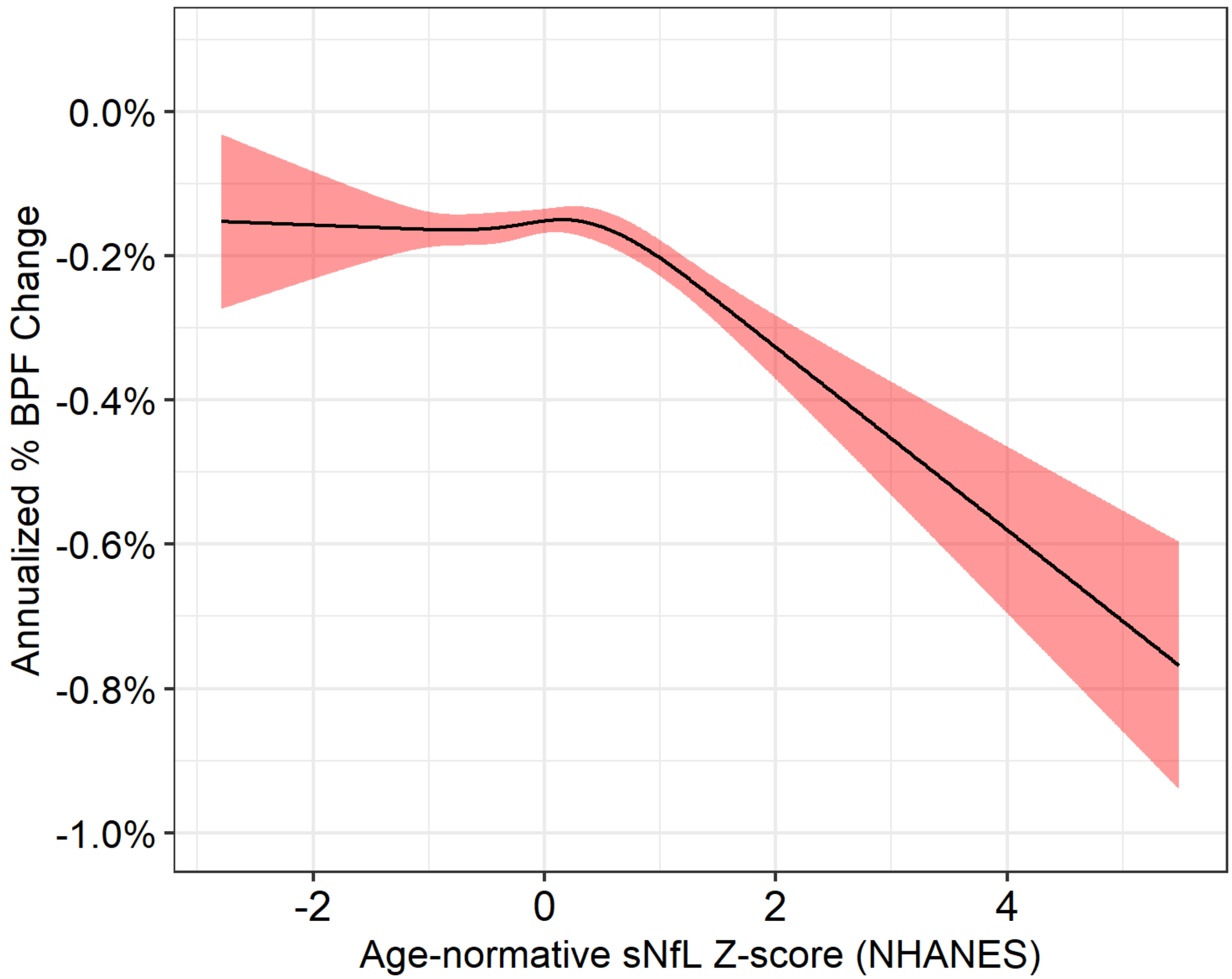

Relationship of annualized percent change in brain parenchymal fraction (BPF) with age-normative sNfL Z-scores (derived from NHANES) in the MS participants. The estimates were derived from a mixed effects regression model using restricted cubic splines to model the sNfL Z-score (likelihood ratio test compared to linear model $p<0.001$). The solid line corresponds to the estimated mean annualized percent change in BPF, and the bounds of the shaded area to the 95% confidence intervals.
